# Isolates, Antimicrobial Susceptibility Profiles and Multidrug Resistance of Bacteria Cultured from Pig Submissions in New Zealand

**DOI:** 10.3390/ani10081427

**Published:** 2020-08-14

**Authors:** Christopher B. Riley, Kirsty L. Chidgey, Janis P. Bridges, Emma Gordon, Kevin E. Lawrence

**Affiliations:** 1 School of Veterinary Science, Massey University, Palmerston North 4442, New Zealand; bridges@xtra.co.nz (J.P.B.); e.gordon1@massey.ac.nz (E.G.); K.Lawrence@massey.ac.nz (K.E.L.); 2 School of Agriculture and Environment, Massey University, Palmerston North 4442, New Zealand; K.L.Chidgey@massey.ac.nz

**Keywords:** antimicrobial, resistance, multidrug, bacteria, susceptibility, pig, pork, porcine

## Abstract

**Simple Summary:**

Data on the bacterial pathogens and the frequency of antimicrobial resistance (AMR) in New Zealand’s pork industry are limited. This study describes bacterial isolates, antimicrobial susceptibility data, and multidrug resistance (MDR; resistance to ≥3 antimicrobial classes) from New Zealand pig submissions. Porcine bacterial culture test results from June 2003 to February 2016 were obtained from commercial veterinary pathology laboratory records. In total, 470/477 unique submissions resulted in bacterial growth, yielding 779 isolates. Sample type was recorded for 75.5%; lung (21.9%), faecal (16.9%) and intestinal (12.5%) were most common. The most common isolates were *Escherichia coli* (23.9%), *Actinobacillus pleuropneumoniae* (5.5%), *Streptococcus suis* (5.5%), unidentified *Campylobacter* spp. (4.9%), alpha hemolytic *Streptococci* (4.1%), coagulase negative *Staphylococcus* spp. (3.3%), and *Pasteurella multocida* (3.2%). Susceptibility results were available for 141/779 (18.1%) isolates from 62/470 (13.2%) submissions. Most were susceptible to trimethoprim-sulphonamide (92.6%), but fewer were susceptible to penicillin (48.1%), tilmicosin (41.9%), or tetracyclines (36.0%). No susceptibility data were for available *Salmonella* spp., *Campylobacter* spp., or *Yersinia* spp. isolates. MDR occurred in 42.6% of tested isolates. Data on sample submission drivers, antimicrobial drug use, and susceptibilities of important porcine bacterial isolates are required to inform guidelines for prudent antimicrobial use, to reduce their prevalence and MDR.

**Abstract:**

Data on the scope of bacterial pathogens present and the frequency of antimicrobial resistance (AMR) in New Zealand’s pigs are limited. This study describes bacterial isolates, antimicrobial susceptibility data, and multidrug resistance (MDR; resistance to ≥3 antimicrobial classes) from New Zealand pig submissions. Porcine test data from June 2003 to February 2016 were obtained from commercial veterinary pathology laboratory records. In total, 470/477 unique submissions resulted in bacterial growth, yielding 779 isolates. Sample type was recorded for 360/477 (75.5%); lung (79/360; 21.9%), faecal (61/360; 16.9%) and intestinal (45/360; 12.5%) were most common. The most common isolates were *Escherichia coli* (186/779, 23.9%), *Actinobacillus pleuropneumoniae* (43/779; 5.5%), *Streptococcus suis* (43/779; 5.5%), unidentified *Campylobacter* spp. (38/779; 4.9%), alpha haemolytic *Streptococci* (32/779; 4.1%), coagulase negative *Staphylococcus* spp. (26/779; 3.3%), and *Pasteurella multocida* (25/779; 3.2%). Susceptibility results were available for 141/779 (18.1%) isolates from 62/470 (13.2%) submissions. Most were susceptible to trimethoprim-sulphonamide (75/81; 92.6%), but fewer were susceptible to penicillin (37/77; 48.1%), tilmicosin (18/43; 41.9%), or tetracyclines (41/114; 36.0%). No susceptibility data were available for *Salmonella* spp., *Campylobacter* spp., or *Yersinia* spp. isolates. MDR was present in 60/141 (42.6%) isolates. More data on sample submission drivers, antimicrobial drug use, and susceptibilities of important porcine bacterial isolates are required to inform guidelines for prudent antimicrobial use, to reduce their prevalence, human transmission, and to minimise AMR and MDR.

## 1. Introduction

Increased recognition of the consequences of antimicrobial resistance (AMR) in humans and animals has led to monitoring and surveillance programs in many countries, often specific to the species being monitored [1]. They range in complexity from highly structured and regulated systems to those that are passive or reactive. Except for a few species of public health interest such as *Salmonella* spp. [2], current AMR surveillance for bacterial isolates recovered from food animal species in New Zealand falls within the latter approach, relying upon the monitoring of abattoir samples for public health purposes, without direct surveillance of livestock populations. Such surveillance may be more critical within intensive food animal sectors, such as the pig industry, where the use of antibiotics within feedstuffs [3,4] may contribute to levels of antimicrobial resistance in clinically ill and healthy animals [5,6,7].

The size of the commercial pig population in New Zealand is modest by international standards. It includes approximately 100 commercial farms with an average herd size of 300 sows from which 645,900 offspring were weaned in 2018 [8,9]. There are approximately an additional 7000 small pig holdings including the descendants of a small number of domestic Asiatic pigs introduced in the 19th century, now called the New Zealand Kune Kune [10,11]. Despite the relatively small size of the New Zealand pork industry, data on the pathogenic and opportunistic bacterial species affecting these animals is limited [12,13,14,15]. An understanding of the bacterial pathogens and the frequency of AMR of isolates from the national pig population is currently limited in scope and recency [4,16,17].

Veterinarians and their clients may submit samples for bacterial culture and antimicrobial susceptibility testing as part of their herd health management practice, or in response to clinical morbidity and mortality. In New Zealand, some organisms of public health interest are submitted for further evaluation and recording in a centrally managed national database [18]. However, the arising porcine AMR data for most organisms are not. Notwithstanding the limitations of interpreting these data within the context of the national pig population, the systematic collation of data generated from these submissions may contribute to the passive surveillance of AMR in the pork industry [19]. This approach may also be of assistance in underpinning relevant antimicrobial use policies [20], and for understanding the risk that bacterial pathogens and AMR may pose to public health and New Zealand’s pork industry. The purpose of this study is to describe bacterial isolates cultured from porcine laboratory submissions, and proportions of cultured bacterial isolates identified as expressing antimicrobial and multidrug resistance in New Zealand.

## 2. Materials and Methods

Results from the bacterial culture of porcine sample submissions from June 2003 to February 2016 and antimicrobial susceptibility test data for isolates were obtained from five commercial veterinary pathology laboratories located in Auckland, Hamilton, Palmerston North, Christchurch, and Dunedin (Gribbles Veterinary, Healthscope©, Australia and New Zealand). Data obtained included the submission date, accession number, the signalment of the pig from which the sample was collected, the region within New Zealand from where the sample originated, a specimen description, the bacterial isolates cultured and, if performed, the antimicrobial panel susceptibilities of the isolates. Antimicrobial susceptibility testing was performed using disk diffusion assays [21]. Data identifying the owners or farms from which samples were submitted collected were not available. 

Bacterial culture and susceptibility results of all submissions with the sampled species identified as a pig or listed as a porcine breed were evaluated irrespective of age or sex. Data not confidently classified as porcine-related were excluded. Specimen descriptions for the samples, stating either the sample type or the anatomical origin were inconsistent. Therefore, they were grouped into broader categories based on anatomical regions or organ systems (e.g., gut and small intestine were grouped within intestinal).

The number of each bacterial species isolated was determined where species data was available, or by genus when the species was not determined. For each species (or genus) the number and proportion of isolates tested for antimicrobial susceptibility were also determined. To reflect changes in nomenclature during the time spanned by the data, organisms grouped as *Trueperella pyogenes* included those listed as *Actinomyces pyogenes* or *Arcanobacterium pyogenes*. Bacteria listed as *Actinobacillus pleuropneumoniae* included those recorded as *Haemophilus pleuropneumoniae.*

Organisms recorded as demonstrating marginal or intermediate susceptibility, or as resistant to a tested antimicrobial, were classed as resistant (i.e., not susceptible). An isolate not susceptible to an antimicrobial agent was considered as having AMR to that compound. Bacterial isolates reported as resistant to one agent in three or more antimicrobial classes were identified as multidrug resistant (MDR) [22]. Antimicrobials and their classes identified for determining AMR and MDR respectively included β-lactams (oxacillin, penicillin and cephalosporins (cephalexin, cephalothin, ceftazidime and ceftiofur)), aminoglycosides (apramycin, gentamicin, neomycin, streptomycin, and spectinomycin), fluoroquinolones (enrofloxacin and marbofloxacin); lincosamides (clindamycin and lincomycin), and macrolides (erythromycin, tilmicosin and tylosin). Antimicrobials that had only a single drug tested within their class included chloramphenicol, fusidic acid, polymixin B, tetracycline, and trimethoprim-sulphonamide.

Summative and comparative analyses were undertaken in Excel (Microsoft Excel^®^, Version 16.21.19012303, Microsoft Corporation, Redmond, WA, USA). Ages of the animals were described numerically for some pigs and categorically for others. For the latter ages were transformed to days, and the median and interquartile range calculated.

## 3. Results

After data screening as described, there were 477 unique porcine laboratory submissions for bacterial culture between June 2003 to February 2016. Of these, 470/477 (98.5%) yielded bacterial growth producing 779 isolates. Of these, 62/470 (13.2%) submissions producing 144 isolates had antimicrobial susceptibility testing performed. 

### 3.1. Signalment and Submission Data

Signalment data within the databases was inconsistently described in the databases. Breeds described in descending order of frequency were Large White (*n* = 59/477; 12.4%) or Large White cross (*n* = 8/477; 1.7%), Auckland Island (*n* = 44/477; 9.2%) or Mixed breed (*n* = 7/477; 1.5%), Kune Kune (*n* = 5/477; 1.0%), Landrace (*n* = 2/477; 0.4%) or Landrace cross (*n* = 3/477; 0.6%), Duroc (*n* = 2/477; 0.4%), Berkshire (*n* = 1/477; 0.2%) or Saddleback (*n* = 1/477; 0.2%). Breed was not recorded or unknown for 345/477 (72.3%) submissions. Records described sex as female (*n* = 72/477; 15.1%), male (*n* = 66/477; 13.8%), mixed (*n* = 43/477; 9%), or unknown or not recorded (*n* = 296/477; 62.1%). Submission ages were listed either in days (*n* = 46/477), weeks (*n* = 104/477), months (35/477), years (*n* = 29/477), or categorically (*n* = 263/477). The ages of pigs that were listed numerically (214/477; 44.9%) has a median age of 56 days (interquartile range 90 days; min 0 days; max 11 years). The remaining records described the age of the pig as foetus (*n* = 2/477; 0.4%), neonate (*n* = 4/477; 0.8%), young (*n* = 12/477; 2.5%), adult or mature (*n* = 6/477; 1.3%), mixed (*n* = 25/477; 5.2%), or unknown (*n* = 214/477; 44.9%). The animal’s age was not described (unknown) for 214/477 (44.9%) records.

**Figure 1:**
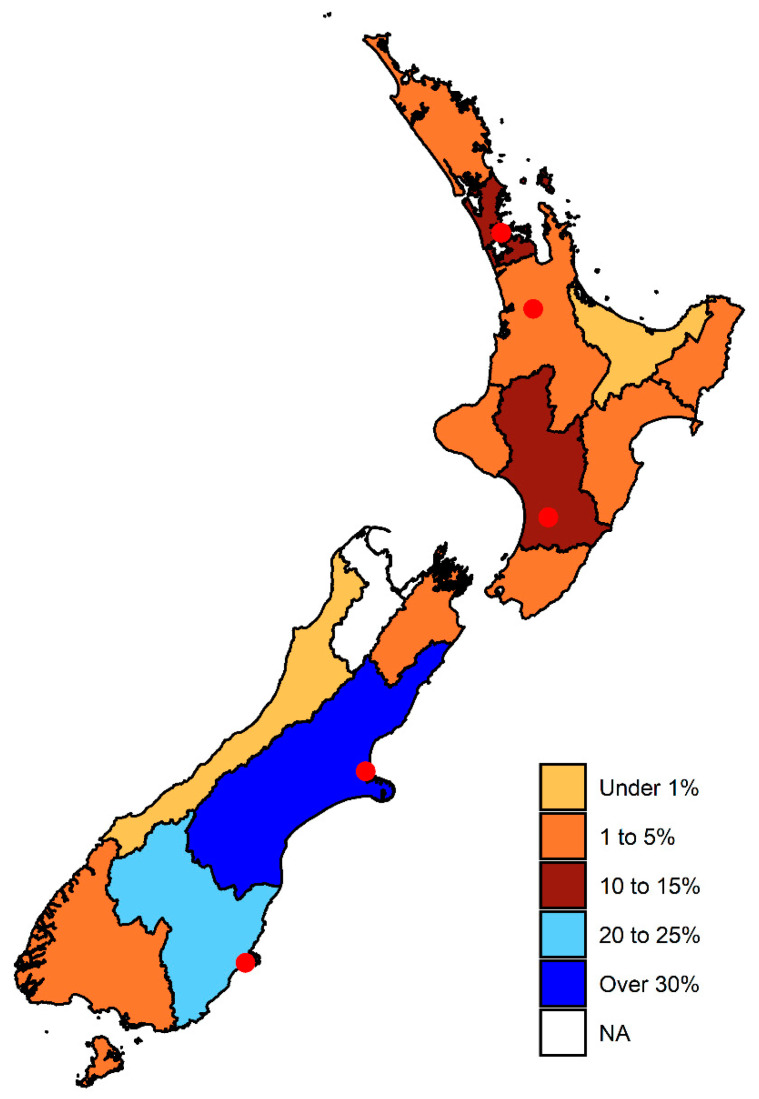
Percentage of submissions (*n* = 475) submitted for bacterial culture and antimicrobial susceptibility testing to commercial veterinary diagnostic laboratories between 2003 and 2016, from pigs in different regions of New Zealand as demarcated on the map. The locations of veterinary laboratories where porcine samples were submitted are indicated as red circles.

Most submissions were received by the Christchurch laboratory (269/477; 50.4%), followed by Palmerston North (93/477; 19.5%), Auckland (86/477; 18.0%), Dunedin (24/477; 5.0%), and Hamilton (5/477; 1%) (Figure 1). In decreasing order the number of samples submitted by region were from Canterbury (146/477; 30.6%), Otago (108/477; 22.6%), Auckland (70/477; 14.7%), Manawatu-Wanganui (53/477; 11.1%), Taranaki (20/477; 4.2%), Southland (19/477; 4.0%), Hawke’s Bay (15/477; 3.1%), Marlborough (13/477; 2.7%), Northland (13/477; 2.7%), Waikato (6/477; 1.3%), Wellington (5/477; 1.0%), Gisborne (5/477; 1.0%), Bay of Plenty (1/477; 0.2%), and the West Coast (1/477; 0.2%). The region from which the sample was submitted was not recorded for 2/477(0.4%) samples.

Only 360/477 (75.5%) of submissions recorded a sample type or anatomic origin. The most common submitted specimens were lung (79/360; 21.9%), faecal (61/360; 16.9%), intestinal (45/360; 12.5%), liver (13/360, 3.6%), stomach (11/360; 3.1%) and semen (11/360; 3.1%). Most submissions were from a single anatomic location or specimen (315/360; 87.5%), 8/360 (2.2%) from multiple gastrointestinal sites, and 37/360 (10.3%) were from multiple sites or organs (range 2 to 6 sites or tissues). There were 64/360 (17.8%) swabs and 25/360 (6.9%) tissues submitted without an identified source or origin. Sample sites for the seven most common bacterial isolates cultured from porcine samples for the period 2003 to 2016 are listed in Table 1.

### 3.2. Culture Results from Submissions

The numbers of isolates from submissions identified by species (*n* = 57 species including staphylococcal and streptococcal subtypes (*n* = 7)) and those characterised to the genus level (*n* = 24 genera) are listed in Table 2. Including staphylococcal and streptococcal subtypes, 539/779 (69.2%) isolates were identified by species, 139/779 (17.8%) by genus only, and the remainder (101/779; 13%) were not identified by species or genus (e.g., mixed bacteria; coliforms; mixed normal flora; etc.) (Table 2). The most commonly identified isolates were *Escherichia coli* (186/779, 23.9% for all strains), followed in order of decreasing frequency by *Actinobacillus pleuropneumoniae*, *Streptococcus suis*, other *Campylobacter* spp., alpha hemolytic *Streptococci*, coagulase negative *Staphylococcus* spp. and *Pasteurella multocida* (Table 2). *Salmonella* spp. (12/779; 1.5%), *C. coli* and *C. jejuni* (29/779:3.7%), and *Yersinia* spp. (9/779; 1.2%) were infrequently isolated. 

### 3.3. Antimicrobial Susceptibility and Multidrug Resistance

The number of antimicrobials each isolate was tested against ranged from 2 to 11 (median 7). For submissions where antimicrobial susceptibility testing was performed, the median number of isolates tested per sample was 2 (range 1–9). Interpretable susceptibility results were available for 141/779 (18.1%) isolates from 62/470 (13.2%) submissions (Table 2). Three organisms (3/144; 3/779) that were submitted for susceptibility testing did not have interpretable results in the database (results were recorded as “~DEL”). No antimicrobial susceptibility data were available for isolates of *Salmonella* spp., *Campylobacter* spp., or *Yersinia* spp. Overall, most tested isolates were susceptible to trimethoprim-sulphonamide (75/81; 92.6%), but fewer isolates were susceptible to penicillin (37/77; 48.1%), tilmicosin (18/43; 41.9%), or tetracyclines (41/114; 36.0%).

There were 60/141 (42.6%) isolates tested for antimicrobial susceptibility that demonstrated MDR (i.e., resistance to ≥3 antimicrobial classes) (Table 3). Sample sizes within bacterial species were small, but where the same species of the organism was cultured from four or more submissions, MDR was a more frequent finding for *Streptococcus suis,* alpha-haemolytic and beta-haemolytic *Streptococci*, and *Corynebacterium* spp.

## 4. Discussion

This study provides a broader overview of the bacterial species associated with infectious disease in New Zealand pigs than has previously been published [4,16,17]. The distribution of isolates was biased towards bacterial pathogens associated with enteric disease, respiratory disease, meningitis and sudden death [15,23,24]. Although the size of the study population was small, the percentage of isolates expressing MDR was generally higher than that recently reported for other farm animal species in New Zealand [25,26]. The limited number of sample submissions from this population is at odds with estimates of antimicrobial sales in the pork industry, indicating that, in common with other industry sectors, factors other than culture and susceptibility results are the main drivers of antimicrobial use in New Zealand [4,27,28].

*E. coli* was the most common bacterial species identified within this population, but only a quarter of these isolates (141/186) were serotyped, and of these, half were positive for the K88 adhesin associated with postweaning diarrhoea in pigs [29]. Peer-reviewed publications describing the pathogenicity of porcine strains of *E. coli* in New Zealand are scant [4,30,31]. Effective vaccination may be used as one of the strategies to prevent porcine *E. coli* diarrhoea but requires serotyping to identify suitable vaccinate strains [29]. Evidence of subtyping in the database studied was limited, and there was no information on how the culture results were utilised by submitting veterinarians. It is recommended that isolates are routinely serotyped for differentiation of strains of differing pathogenicity in pigs, and to identify those of public health significance [30,31].

*A. pleuropneumoniae*, the second most commonly cultured isolate, is widespread in New Zealand’s pigs, and is an important cause of pleuropneumonia and septicaemia [3,13,32]. The current true prevalence in the pig population is unknown, but given the frequency of animal movement between large commercial and small-scale piggeries [10], it is possible that the 5.5% of isolates is associated with an increase in prevalence from 2.7% of porcine abattoir samples found in 1998 [3,13]. The isolation of *S. suis* from New Zealand pigs was first described in the 1980s and is associated with neurologic and arthritic disease [33]. In addition to its importance as a pig pathogen, the organism is a zoonosis of emerging importance, with farmers, food processors and veterinarians at risk [34,35]. A high prevalence of serotypes 1 and 2 was found in the New Zealand pig population in the 1980s, but there are no contemporary published data [33]. In the current data set, most isolates (95.3%) were not typed. However, serotyping is critical in understanding the epidemiology and pathogenesis of the *S. suis* in pigs, as well as its pathogenicity as a zoonotic agent [35,36].

*Campylobacter* spp. were also frequently isolated. *C. coli* is considered the dominant species in pigs [24], but in the current study, *C. jejuni* isolates were more common. However, most *Campylobacter* spp. isolates (55.9%) were not speciated, limiting further conclusions. Similar numbers of each species were isolated in a study of pig offal in New Zealand [17], but *C. jejuni* is considered to be of greater public health significance [37,38]. *Salmonella* spp. and *Yersinia* spp. were infrequently isolated. Although pigs are routinely targeted for surveillance to detect these organisms at abattoirs [18], the low numbers of *Salmonella* spp. isolates in the current study are consistent with low numbers in recent governmental animal health surveillance reports for pigs [39]. 

Although fewer than 20% of isolates from 13% of submissions were tested for antimicrobial susceptibility, these rates are markedly higher than those reported in contemporary New Zealand studies of beef and preproduction cattle (9.2% and 6.6% respectively), and sheep (5.3% and 2.5% respectively) [26]. A recent report in the New Zealand dairy industry showed that 21% of veterinarians utilise susceptibility testing, but it is unclear if this corresponds with the number of submissions for bacterial culture [28]. The low level of susceptibility testing is concerning as it further emphasises a disparity between susceptibility-guided prudent antimicrobial use in the livestock industries in New Zealand and annual antimicrobials sales [4,25,27]. Evidence for New Zealand livestock veterinarians is scant, but a recent survey found that 80% of dairy veterinarians prescribe antimicrobials based on a diagnosis, and 65% as a test for response to therapy, in lieu of bacterial culture and antimicrobial susceptibility testing [28]. For some pathogenic organisms, the identification of species may direct other control measures, such as vaccination (e.g., *E. coli*) or providing zinc oxide in feed at weaning [40], rather than antimicrobial use [23,29]. Given the low submission rate for pigs and lack information on how these laboratory results are utilised, closer scrutiny of the drivers for sample submission and antimicrobial use is recommended. 

Interpretation of the significance of antimicrobial susceptibility results is limited by the small number of isolates within each species or genus grouping tested, and the use of different antimicrobial panels for the same bacterial species in the dataset (Table 2). For *E. coli* isolates, the most common isolate tested, susceptibility to tetracycline (25%), was low, but high to neomycin (92%), enrofloxacin (92%), and trimethoprim-sulphonamide (95%). These findings are largely in agreement with a United Kingdom study of 152 isolates that comparable susceptibilities for tetracycline (20%), neomycin (95%) and enrofloxacin (80–93%), but there was a better response to trimethoprim-sulphonamide (55%) [40]. A larger pool of *E. coli* clinical isolates (*n* = 2144) from the United States had a low susceptibility to tetracycline and neomycin (50%), a strong response to enrofloxacin (98%), and moderate susceptibility to trimethoprim-sulphonamide (74%) [40]. A 2001 study of New Zealand 296 *E. coli* isolates from conventionally farmed healthy pigs identified a higher susceptibility to tetracycline (60%), and a similar response to neomycin (99%) (4). Susceptibility to enrofloxacin and trimethoprim-sulphonamide were not tested by Nulsen et al. (2008), but susceptibility to amoxicillin in the current group of submissions (60%) was markedly lower than that previously determined for healthy New Zealand pigs (98.3%) [4].

Antimicrobials are an important tool in combating the bacterial pathogens associated with Porcine Respiratory Disease Complex (PRDC) [41]. *A. pleuropneumoniae* and *P. multocida* are common bacterial pathogens associated with PRDC, and were common isolates cultured in the current study. None of the isolates from these two bacterial species displayed MDR, in agreement with a recent study of these pathogens in Spain [41]. However few isolates within the current database were subjected to susceptibility testing.

As described for other livestock species in New Zealand, susceptibility and MDR data for *Campylobacter* spp., *Salmonella* spp., and *Yersinia* spp. were not available [25]. *Campylobacter coli* and *C. jejuni* isolates from pig offal sources within some regions of New Zealand have a higher level of resistance to erythromycin (35.7%) than other livestock, or human-derived isolates [17]. *Campylobacter* spp. are the second most common zoonoses of public health concern in New Zealand [42], and the lack of AMR for the porcine isolates in this database limits the value of these data for informing prudent use guidelines in the pork industry. The other most common isolates, *A. pleuropneumoniae*, and *S. suis* had so few susceptibility data that meaningful recommendations for antimicrobial use cannot be made.

The total level of MDR (42.6%) was double that reported for a contemporary studies of AMR and MDR in New Zealand beef and preproduction dairy cattle (20.7%) and sheep (20.5%), and 60% greater than the rate for mixed bacterial infections in New Zealand equine neonates (26%) [25,26,43]. Although antimicrobial use practices were not documented in the current study, there is evidence that the oral administration of antimicrobials, as commonly practiced within the pork industry, may be a significant risk factor for AMR and MDR for bacteria recovered from pigs [5]. For individual bacterial species, meaningful comparisons the published literature are limited by small sample size in the current study. However, MDR rates for the more commonly evaluated bacterial species of *E. coli* and coagulase-negative *Streptococci* were moderate.

These data provide an overview of the different bacterial species associated with diseased New Zealand pigs, but in common with recent works in other species, the lack of clinical context driving submission behaviours and the size of the data set limit the broader application of these findings [25,43,44]. Inconsistent and incomplete signalment data and nonstandardised approaches to susceptibility testing during the period of review prevented the use of epidemiologic modelling to identify risk factors for AMR. Accurate signalment information, culture, and antimicrobial susceptibility results underpin meaningful laboratory interpretation, and effective AMR surveillance [45,46]. The applicability of the current findings across the New Zealand pork industry is limited due to the low numbers of isolates submitted for antimicrobial susceptibility testing. Furthermore, the frequency of AMR and MDR must be interpreted with caution. Antimicrobial panels used for susceptibility testing did not differentiate between intrinsically resistant isolates and those expressing acquired resistance [47]. For example, Enterobacteriaceae phenotypes include those intrinsically resistant to benzylpenicillin, macrolides, lincosamides, streptogramins, and rifampin [47]. Ideally, antimicrobial panels used for susceptibility testing should be specifically selected for the bacterial isolate tested, using animal-specific CSLI resistance breakpoints [48].

## 5. Conclusions

This is the first report on the breadth of bacterial species, antimicrobial susceptibility and MDR based on a wide spectrum of isolates from laboratory submissions for New Zealand pigs. MDR rates are higher in pigs and those recently reported for other New Zealand livestock species. These findings contribute to an understanding of the scope of bacterial species associated with porcine disease in New Zealand but provide limited information on AMR in this population. The use of bacterial culture and susceptibility testing in the New Zealand pork industry is limited. More data on sample submission behaviours, antimicrobial drug uses in the pork industry, and the susceptibilities of important porcine bacterial isolates are required to inform guidelines for their prudent use in this industry. Data captured by the organised monitoring of bacterial isolates of note or potential public health importance from pigs may inform strategies that reduce their prevalence and transmission of those with zoonotic potential in New Zealand.

## Figures and Tables

**Table 1:** Sample sites for the seven most common bacterial isolates cultured from porcine samples. The percentage of total isolates for each isolate type by sampling site are shown in parentheses.

	Species of Bacterial Isolates							
Sample Type ^a^	*Escherichia coli*	*Actinobacillus pleuropneumoniae*	*Campylobacter* species	*Pasteurella multocida*	*Stretocoocus suis*	*Staphylococci* Coagulase-Negative	*Streptococci* alpha Haemolytic	Total for all Isolates
Eye swab	1/98 (1.0)	-	-	-	-	-	-	1/360 (0.3)
Upper respiratory	-	-	-	-	-	-	-	3/360 (0.8)
Lung	10/98 (10.2)	29/35 (82.9)	-	8/15 (53.3)	4/28 (14.3)	1/4 (25)	-	79/360 (21.9)
Heart or pericardial fluid	-	-	-	-	1/28 (3.6)	-	-	5/360 (1.4)
Abdominal fluid	-	-	-	-	2/28 (7.1)	-	-	1/360 (0.3)
Stomach	4/98 (4.1)	-	-	-	-	-	1/6 (16.7)	11/360 (3.1)
Intestinal	22/98 (22.4)	-	9/26 (34.6)	-	1/28 (3.6)	-	-	45/360 (12.5)
Colon, caecum or rectal	2/98 (2.0)	-	2/26 (7.7)	-	-	-	-	7/360 (1.9)
Faecal	30/98 (30.6)	-	14/26 (53.8)	-	-	-	1/6 (16.7)	61/360 (16.9)
Liver	1/98 (1.0)	-	-	1/15 (6.7)	1/28 (3.6)	-	1/6 (16.7)	13/360 (3.6)
Spleen	-	-	-	-	2/28 (7.1)	1/4 (25)	-	4/360 (1.1)
Kidney	-	-	-	-	1/28 (3.6)	-	-	3/360 (0.8)
Lymph node	1/98 (1.0)	-	-	-	-	-	-	3/360 (0.8)
Female urogenital	-	-	-	-	-	-	-	6/360 (1.7)
Semen	3/98 (3.1)	-	-	-	-	-	-	11/360 (3.1)
Urine	2/98 (2.0)	-	-	-	-	-	-	3/360 (0.8)
Skin	1/98 (1.0)	-	-	-	1/28 (3.6)	-	-	4/360 (1.1)
Foot	-	-	-	-	-	-	-	2/360 (0.6)
Tissue	3/98 (3.1)	1/35 (2.9)	1/26 (3.8)	3/15 (20.0)	8/28 (28.6)	1/4 (25)	-	25/360 (6.9)
Swab	18/98 (18.4)	5/35 (14.3)	-	3/15 (20.0)	7/28 (25.0)	1/4 (25)	2/6 (33.3)	64/360 (17.8)
Abscess	-	-	-	-	-	-	-	4/360 (1.1)
Fluid	-	-	-	-	-	-	-	1/360 (0.3)
Milk	-	-	-	-	-	-	-	1/360 (0.3)
Aspirate	-	-	-	-	-	-	1/6 (16.7)	1/360 (0.3)
Blood culture	-	-	-	-	-	-	-	1/360 (0.3)
Transport media	-	-	-	-	-	-	-	1/360 (0.3)

^a^ Sample type or site was recorded for only 360/477 (75.5%) submissions.

**Table 2:** Bacterial species cultured from New Zealand porcine samples submissions and proportion tested for antimicrobial susceptibility.

Bacterial Species	Number of IsolatesCultured (%) ^a^		Proportion Tested forAntimicrobial Susceptibility ^b^ (%)	
*Acinetobacter johnsonii*	1	(0.1)	0	(0)
*Actinobacter lwoffii*	2	(0.3)	2/2	(50)
*Actinobacter* species	9	(1.2)	5/9	(44.4)
*Actinobacillus pleuropneumoniae*	43	(5.5)	2/43	(4.7)
*Actinobacillus* species	4	(0.5)	0	(0)
*Aeromonas* species	2	(0.3)	0	(0)
*Aeromonas veronii*	1	(0.1)	0	(0)
*Alcaligenes species*	1	(0.1)	0	(0)
*Arcanobacterium haemolyticum*	1	(0.1)	0	(0)
*Arcanobacterium* species	1	(0.1)	0	(0)
*Bacillus* species	3	(0.4)	1/3	(33.3)
*Bacteroides* species	4	(0.5)	1/4	(25)
*Bordetella bronchiseptica*	2	(0.3)	0	(0)
*Brachyspira (Serpulina) hyodysenteriae*	1	(0.1)	0	(0)
*Brachyspira pilosicoli*	2	(0.3)	0	(0)
*Burkholderia cepacian*	4	(0.5)	3/4	(50)
*Campylobacter coli*	8	(1.0)	0	(0)
*Campylobacter jejuni*	14	(1.8)	0	(0)
*Campylobacter jejuni/coli*	7	(0.9)	0	(0)
*Campylobacter* species	38	(4.9)	0	(0)
*Campylobacter upsaliensis/helveticus*	1	(0.1)	0	(0)
*Citrobacter freundii*	1	(0.1)	0	(0)
*Citrobacter species*	2	(0.3)	1/2	(50)
*Clostridium perfringens*	7	(0.9)	0	(0)
*Corynebacterium* species	14	(1.8)	4/14	(28.6)
*Enterobacter* species	1	(0.1)	0	(0)
*Enterococcus faecalis*	11	(1.4)	5/11	(45.5)
*Enterococcus* species	11	(1.4)	1/11	(9.1)
*Erysipelothrix rhusiopathiae*	1	(0.1)	0	(0)
*Escherichia coli*	107	(13.7)	26/107	(24.3)
*Escherichia coli* (K88 negative)	20	(2.6)	2/20	(10)
*Escherichia coli* (K88 positive)	23	(3.0)	0	(0)
*Escherichia coli -* beta haemolytic	32	(4.1)	4/32	(12.5)
*Escherichia coli -* beta haemolytic (K88 negative)	3	(0.4)	0	(0)
*Escherichia coli -* beta haemolytic (K88 positive)	1	(0.1)	0	(0)
*Fusobacterium* species	1	(0.1)	0	(0)
*Haemophilus parasuis*	3	(0.4)	1/3	(33.3)
*Klebsiella oxytoca*	2	(0.3)	2/2	(100)
*Klebsiella pneumoniae*	4	(0.5)	3/4	(75)
*Klebsiella* species	2	(0.3)	0	(0)
*Lactobacillus* species	1	(0.1)	0	(0)
*Micrococcus* species	2	(0.3)	2/2	(100)
*Moraxella* species	1	(0.1)	0	(0)
*Morganella morganii*	1	(0.1)	1/1	(100)
*Mycoplasma* species	1	(0.1)	0	(0)
*Pasteurella multocida*	25	(3.2)	4/25	(16)
*Pasteurella pneumotropica*	2	(0.3)	1/2	(50)
*Pasteurella* species	3	(0.4)	1/3	(33.3)
*Proteus mirabilis*	8	(1.0)	3/8	(37.5)
*Proteus* species	9	(1.2)	0	(0)
*Proteus vulgaris*	1	(0.1)	0	(0)
*Providencia stuartii*	2	(0.3)	1/2	(50)
*Pseudomonas aeruginosa*	8	(1.0)	4/8	(50)
*Pseudomonas fluorescens*	1	(0.1)	0	(0)
*Pseudomonas* species	8	(1.0)	2/8	(25)
*Salmonella* Brandenburg	1	(0.1)	0	(0)
*Salmonella* species	4	(0.5)	0	(0)
*Salmonella* Tennessee	1	(0.1)	0	(0)
*Salmonella* Typhimurium	6	(0.8)	0	(0)
*Serratia marcescens*	2	(0.3)	1/2	(50)
*Staphylococcus aureus*	11	(1.4)	4/11	(36.4)
*Staphylococcus hyicus*	10	(1.3)	3/10	(30)
*Staphylococcus intermedius*	3	(0.4)	0	(0)
*Staphylococcus* species	8	(0.1)	1/8	(12.5)
*Staphylococcus* species - coagulase negative	26	(3.3)	14/26	(53.8)
*Streptococcus bovis*	1	(0.1)	1/1	(100)
*Streptococcus dysgalactiae*	5	(0.6)	3/5	(60)
*Streptococcus equi subsp. zooepidemicus*	2	(0.3)	0	(0)
*Streptococcus porcinus*	2	(0.3)	½	(50)
*Streptococcus suis* ^c^	43	(5.5)	4/43	(9.3)
*Streptococcus viridans*	1	(0.1)	1/1	(100)
*Streptococcus* Lancefield Group A	1	(0.1)	0	(0)
*Streptococcus* Lancefield Group C	10	(1.3)	5/10	(50)
*Streptococcus* Lancefield Group D	3	(0.4)	2/3	(66.7)
*Streptococcus* species	8	(1.0)	3/8	(37.5)
*Streptococci*-alpha haemolytic	32	(4.1)	6/32	(18.8)
*Streptococci*-beta haemolytic	10	(1.3)	3/10	(30)
*Streptococci*-non-haemolytic	3	(0.4)	1/3	(33.3)
*Trueperella pyogenes*	8	(1.0)	1/8	(12.5)
*Yersinia enterocolitica*	1	(0.1)	0	(0)
*Yersinia pseudotuberculosis*	7	(0.9)	0	(0)
*Yersina* species	1	(0.1)	0	(0)
Other not identified to a genus or species	101	(13.0)	8/101	(7.9)
Total susceptibility tested	779	(100)	144/779	(18.5) ^d^

^a^ 779 isolates from 470 submissions; ^b^ 144 isolates from 62 submissions; ^c^ One isolate was type 1, one was type 2, and the remainder were not subtyped; ^d^ Three organisms (3/144; 3/779) that were submitted for susceptibility testing did not have interpretable results in the record.

**Table 3:** Antimicrobial susceptibility and multidrug resistance for tested bacteria isolated from submissions for New Zealand pigs for the period June 2003 to February 2016.

		Antimicrobial Used For Testing Susceptibility ^a,b^																					
Bacterial Species	AMX	AMC	CEPX	CEPH	CEFT	OXA	PEN	APR	GEN	NEO	SPEC	STR	ENR	MAR	ERY	TIL	CLI	LNC	TET	TMS	CHL	POLY	MDR (%) ^c^
*Actinobacter lwoffii*	-	1/2	1/2	-	1/2	-	1/2	-	1/2	1/2	-	1/2	0/1	-	-	-	-	-	-	-	-	-	0/2 (0)
*Actinobacter* specie	2/3	0/2	-	2/3	2/4	-	0/3	2/3	4/5	0/3	2/2	3/4	3/4	-	-	1/1	0/2	0/1	3/4	3/3	-	1/1	1/6 (16.7)
*Actinobacillus pleuropneumoniae*	2/2	-	-	2/2	2/2	-	2/2	-	-	-	1/1	-	1/1	-	1/1	1/1	2/2	-	2/2	2/2	-	-	0/2 (0)
*Bacillus* species	1/1	-	-	-	-	-	1/1	-	-	-	-	0/1	0/1	-	-	-	-	-	0/1	1/1	-	-	1/1 (100)
*Bacteroides* species	-	1/1	-	1/1	1/1	-	-	1/1	-	1/1	-	-	-	-	-	-	-	-	0/1	1/1	-	-	0/1 (0)
*Burkholderia cepacia*	1/2	2/3	-	-	1/1	-	0/2	0/1	1/2	2/3	0/1	1/1	1/2	-	-	0/2	0/1	-	1/2	-	0/2	1/1	2/3 (66.7)
*Citrobacter* species	0/1	0/1	-	-	-	-	-	-	-	-	-	0/1	1/1	-	-	-	-	-	1/1	-	1/1	-	0/1 (0)
*Corynebacterium* species	1/1	4/5	1/1	-	2/2	-	2/4	1/3	3/4	4/5	1/2	1/1	3/4	-	0/1	0/1	0/2	0/1	0/3	-	0/2	2/2	3/4 (75)
*Enterococcus faecalis*	2/3	4/5	-	1/3	2/2	0/1	4/5	0/1	0/1	0/1	1/2	-	1/2	1/1	1/2	1/4	0/3	1/1	0/5	2/3	0/1	1/1	3/5 (60)
*Enterococcus* species	0/1	0/1	-	-	-	-	-	-	-	-	-	0/1	1/1	-	-	-	-	-	0/1	1/1	-	-	1/1 (100)
*Escherichia coli*	9/10	7/18	-	4/5	2/3	-	2/7	4/5	3/4	8/8	4/9	6/9	8/9	-	3/6	2/7	1/7	1/4	6/18	13/14	0/1	2/2	9/26 (34.6)
*Escherichia coli* - beta haemolytic	3/3	3/4	-	-	-	-	-	1/1	-	1/2	0/1	2/2	3/3	-	-	-	-	-	0/4	4/4	-	-	1/4 (25)
*Escherichia coli* (K88 negative)	-	0/1	-	1/1	-	-	-	1/1	-	2/2	-	-	1/1	-	-	-	-	-	0/2	1/2	-	-	1/2 (50)
*Haemophilus parasuis*	1/1	-	-	-	-	-	1/1	-	-	-	-	1/1	1/1	-	-	-	-	-	1/1	1/1	-	-	0/1 (0)
*Klebsiella oxytoca*	1/1	0/1	-	-	-	-	0/1	0/1	0/1	0/1	0/1	-	1/1	-	-	0/1	0/1	-	0/1	-	0/1	1/1	2/2 (100)
*Klebsiella pneumoniae*	1/1	2/4	-	-	1/1	-	0/1	1/1	2/2	2/2	-	3/3	-	-	1/2	1/1	0/2	-	1/3	1/1	-	-	1/3 (33.3)
*Micrococcus* species	1/1	-	-	-	1/1	-	-	1/1	1/1	1/1	-	0/1	-	-	-	0/1	-	-	-	-	-	-	0/1 (0)
*Morganella morganii*	-	1/1	-	-	-	-	0/1	-	1/1	-	-	0/1	1/1	-	-	-	-	0/1	1/1	1/1	-	-	1/1 (100)
*Pasteurella multocida*	2/2	4/4	-	-	-	-	2/2	-	-	-	1/1	0/2	2/2	1/1	1/1	2/2	0/2	-	3/3	4/4	-	-	0/4 (0)
*Pasteurella pneumotropica*	-	1/1	-	1/1	-	-	-	-	-	-	-	0/1	-	-	1/1	1/1	-	1/1	0/1	1/1	-	-	0/1 (0)
*Pasteurella* species	-	1/1	-	2/2	-	-	-	-	-	-	-	1/1	-	-	0/1	1/1	-	-	1/1	1/1	-	-	0/1 (0)
*Proteus mirabillis*	-	3/3	-	1/1	1/1	-	2/2	0/1	1/1	2/2	1/2	-	-	-	1/3	0/1	-	1/3	1/2	2/2	-	-	2/3 (66.7)
*Providencia stuartii*	1/1	-	-	-	1/1	-	0/1	-	1/1	1/1	-	1/1	-	-	-	0/1	-	-	1/1	-	-	-	0/1 (0)
*Pseudomonas aeruginosa*	2/2	2/3	-	1/1	2/2	1/1	0/2	1/2	2/2	2/3	1/2	0/1	0/2	1/1	0/1	0/1	0/1	-	1/2	1/1	-	1/2	2/4 (50)
*Pseudomonas* species	-	0/1	-	0/1	1/1	-	0/2	0/2	1/2	0/2	0/1	0/1	1/2	-	-	-	0/1	-	1/2	1/1	-	1/1	2/2 (100)
*Serratia marcescens*	-	1/1	-	-	1/1	-	0/1	-	1/1	1/1	-	1/1	-	-	-	0/1	-	-	1/1	-	-	-	0/1 (0)
*Staphylococci aureus*	2/2	1/2	-	2/2	-	-	1/2	-	1/1	1/2	-	1/2	0/1	-	2/3	1/1	1/1	1/2	0/4	3/3	-	-	1/4 (25)
*Staphylococcus hyicus*	1/1	3/3	-	1/1	-	-	3/3	-	-	-	1/1	-	-	-	2/3	1/2	2/3	-	0/3	2/2	-	-	1/3 (33.3)
*Staphylococci* - coagulase negative	6/6	8/9	1/1	3/3	4/4	1/1	8/11	2/4	4/4	4/4	5/5	4/6	5/6	1/1	2/4	-	3/8	0/2	6/12	7/7	-	2/2	4/15 (35.7)
*Streptococci* - alpha haemolytic	1/1	3/5	-	1/2	2/2	1/2	0/3	0/1	1/2	0/1	1/1	1/2	1/1	-	1/4	1/2	1/1	0/3	1/5	2/3	-	-	4/6 (66.7)
*Streptococci* - beta haemolytic	1/2	1/1	-	1/2	-	-	0/2	-	1/1	-	-	0/2	2/3	-	-	-	-	0/1	1/3	2/3	-	-	3/4 (75)
*Streptococci* - non-haemolytic	1/1	-	-	-	-	-	0/1	-	-	-	-	0/1	0/1	-	-	-	-	-	0/1	1/1	-	-	1/1 (100)
*Streptococcus bovis*	-	1/1	-	-	-	-	-	-	-	-	-	1/1	-	-	0/1	0/1	-	0/1	1/1	1/1	-	-	0/1 (0)
*Streptococcus dysgalactiae*	2/2	1/1	-	-	-	2/2	1/1	-	-	-	1/1	-	-	-	0/1	1/2	1/1	1/2	1/3	3/3	-	0/1	2/3 (66.7)
*Streptococcus Lancefield Group C*	1/1	5/5	-	2/2	1/1	-	2/3	-	-	2/2	1/2	0/2	-	-	3/5	2/2	1/2	2/2	2/5	5/5	-	-	1/5 (20)
*Streptococcus Lancefield Group D*	-	1/1	2/2	-	-	-	0/1	0/1	1/1	1/2	1/1	-	0/2	-	-	-	0/1	-	0/1	-	-	0/1	2/2 (100)
*Streptococcus porcinus*	-	1/1	-	-	-	-	1/1	-	-	-	-	-	-	-	-	0/1	0/1	-	0/1	-	-	-	1/1 (100)
*Streptococci* species	-	2/3	-	1/1	1/2	-	2/3	0/1	1/2	1/2	-	0/1	1/1	-	0/2	1/1	1/1	0/1	0/1	1/1	-	-	2/3 (66.7)
*Streptococcus suis*	-	4/4	-	-	-	-	1/2	0/1	1/1	0/1	1/1	0/1	0/1	-	2/3	1/3	1/3	0/1	0/4	1/2	-	0/1	3/4 (75)
*Streptococcus viridans*	-	0/1	-	-	-	-	-	-	-	-	-	1/1	-	-	1/1	-	-	0/1	0/1	1/1	-	-	0/1 (0)
*Trueperella pyogenes*	1/1	1/1	-	-	-	-	0/1	0/1	0/1	1/1	0/1	-	0/1	-	-	0/1	0/1	-	0/1	-	0/1	1/1	1/1 (100)
Unidentified bacterial isolates	4/4	2/4	-	-	1/1	-	1/3	0/1	0/1	1/2	0/1	0/1	3/4	-	1/2	-	2/2	0/1	4/5	5/5	-	1/1	2/6 (33.3)

^a^ AMX = amoxicillin and clavulanic acid; AMC = ampicillin; CEPH = cephalothin; CEFT = ceftiofur; CEPX = cephalexin; OXA = oxacillin; PEN = penicillin; APR = aprmycin; GEN = gentamicin; NEO = neomycon; SPEC = spectinomycin; STR = streptomycin; ENR = enrofloxacin; MAR = marbofloxacin; ERY = erythromycin; TIL = tilmicosin; CLI = clindamycin; LNC = lincomycin; TET = tetracycline; TMS = trimethoprim-sulphonamide; CHL = chloramphenicol; POLY = polymixin B. ^b^ Susceptibility to ceftazidime was reported for *E. coli* (1/1 susceptible) and two other unidentified isolates (2/2 susceptible) only; susceptibility to tylosin was reported for *E. coli* (0/1 susceptible) only; susceptibility to fusidic acid was reported for *S. dysgalactiae* (2/2 susceptible) only. ^c^ Proportion and percentage of tested isolates of that species or genus (row) exhibiting multidrug resistance.
